# A critical role of calcineurin in stress responses, hyphal formation, and virulence of the pathogenic fungus *Trichosporon asahii*

**DOI:** 10.1038/s41598-022-20507-x

**Published:** 2022-09-27

**Authors:** Yasuhiko Matsumoto, Asami Yoshikawa, Tae Nagamachi, Yu Sugiyama, Tsuyoshi Yamada, Takashi Sugita

**Affiliations:** 1 Department of Microbiology, Meiji Pharmaceutical University, 2-522-1, Noshio, Kiyose, Tokyo 204-8588 Japan; 2 Teikyo University Institute of Medical Mycology, 359 Otsuka, Hachioji, Tokyo 192-0395 Japan; 3 Asia International Institute of Infectious Disease Control, Teikyo University, 2-11-1, Kaga, Itabashi-ku, Tokyo 173-8605 Japan

**Keywords:** Fungi, Microbial genetics, Pathogens

## Abstract

*Trichosporon asahii* is a conditional pathogenic fungus that causes severe and sometimes fatal infections in immunocompromised patients. While calcineurin, an essential component of a calcium-dependent signaling pathway, is known to regulate stress resistance and virulence of some pathogenic fungi, its role in *T. asahii* has not been investigated. Here, we demonstrated that calcineurin gene-deficient *T. asahii* mutants are sensitive to high temperature as well as cell-membrane and cell-wall stress, and exhibit decreased hyphal formation and virulence against silkworms. Growth of *T. asahii* mutants deficient in genes encoding subunits of calcineurin, *cna1* and *cnb1*, was delayed at 40 °C. The *cna1* and *cnb1* gene-deficient mutants also showed sensitivity to sodium dodecyl sulfate, Congo red, dithiothreitol, and tunicamycin. On the other hand, these mutants exhibited no sensitivity to caffeine, sorbitol, monensin, CaCl_2_, LiCl, NaCl, amphotericin B, fluconazole, or voriconazole. The ratio of hyphal formation in the *cna1* and *cnb1* gene-deficient mutants was decreased. Moreover, the virulence of the *cna1* and *cnb1* gene-deficient mutants against silkworms was attenuated. These phenotypes were restored by re-introducing each respective gene into the gene-deficient mutants. Our findings suggest that calcineurin has a role in regulating the cellular stress response and virulence of *T. asahii*.

## Introduction

*Trichosporon asahii* is a basidiomycete yeast widely distributed in the environment and is often isolated from human blood, sputum, skin, feces, and urine^1–6^. *T. asahii* causes severe fungal infections in immunocompromised patients^7–9^, and the mortality rate of deep mycoses caused by *T. asahii* is twice as high as that caused by *Candida albicans* (80% vs 40%)^10^. *T. asahii* is resistant to echinocandin antifungals, and thus patients treated with micafungin are susceptible to the development of severe infections^11^. Amphotericin B- and azole antifungal-resistant *T. asahii* strains have also been isolated from patients^12,13^. *T. asahii* commonly forms a biofilm comprising microbe aggregates and extracellular matrix on indwelling medical devices^14^. The biofilm formation by *T. asahii* has a function to confer its resistance to antifungal drugs^13^. Several morphologic forms of *T. asahii* exist, such as yeast, hyphae (filament form), and arthroconidia (chains of cells and asexual spores)^4^. Arthroconidia of *T. asahii* contribute to biofilm formation by promoting cellular adhesion^15^. These features of *T. asahii* make it a highly problematic clinical pathogen^9^.

Calcineurin, a calcium-calmodulin-activated phosphatase consisting of a heterodimer with the catalytic and regulatory subunits Cna1 and Cnb1^16^ controls the expression of several genes by dephosphorylating the transcriptional regulator Crz1 through binding to calmodulin, a calcium sensor^17,18^. It is essential for the growth of *Cryptococcus neoformans*, a pathogenic basidiomycete yeast like *T. asahii*, at 37 °C, as well as its virulence in a rabbit model of cryptococcal meningitis and cation homeostasis^19,20^. In addition, calcineurin contributes to resistance to cell membrane damage, cell wall damage, osmotic stress, and endoplasmic reticulum (ER) stress^21,22^. The role of calcineurin in the virulence of *T. asahii*, however, has remained unclear.

To elucidate the virulence of *T. asahii*, we used an established silkworm infection model^23^. Although mammalian experimental models such as mice are usually used in studies of infectious diseases^24^, the requirements of specialized experimental facilities and large numbers of animals as well as the ethical considerations are severely limiting^25^. *T. asahii* infection experiments are also not easy to perform in mice because immunosuppressive drugs must be administered^26,27^. The use of an invertebrate silkworm model for these types of experiments is highly advantageous because silkworms are less costly to house and rear in large numbers, and fewer ethical problems are associated with their use.

In the present study, we used a recently developed technique^28^ to generate calcineurin gene-deficient *T. asahii* mutants and characterized the phenotypes related to stress resistance and virulence in the silkworm infection model. The *cna1* and *cnb1* gene-deficient mutants exhibited sensitivity to chemicals known to cause membrane damage and ER stress, as well as attenuated hyphal formation and virulence against silkworms. Our findings suggest that calcineurin controls cellular stress responses and virulence of *T. asahii* via a calcineurin-signaling pathway.

## Results

### Generation of *cna1*, *cnb1* gene-deficient mutants, and their gene-reintroduced strains

**Figure 1:**
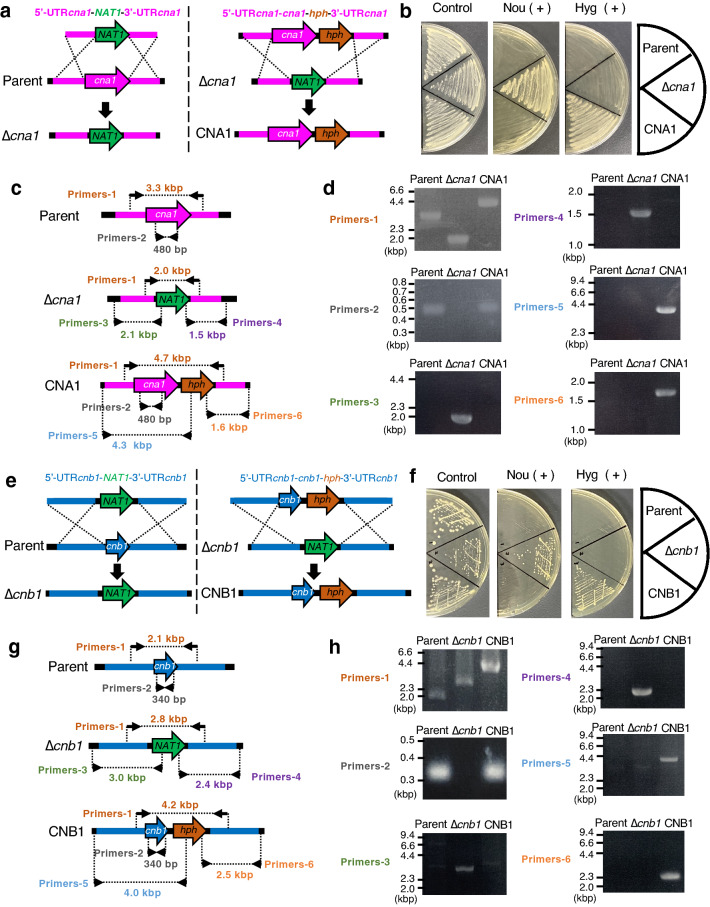
Generation of the *cna1* and *cnb1* gene-deficient *T. asahii* mutants and their revertants. (**a**–**d**) Generation of the *cna1* gene-deficient mutant and its revertant in *T. asahii*. (**a**) Strategy for generating the *cna1* gene-deficient mutant (∆*cna1*) and its revertant (Rev.). Predicted genomes of the *cna1* gene-deficient mutant and its revertant are shown. (**b**) The parent strain (Parent), *cna1* gene-deficient mutant (∆*cna1*), and its revertant (Rev.) were spread on SDA with nourseothricin (Nou) (100 µg/ml) or hygromycin B (Hyg) (100 µg/ml) and incubated at 27 °C for 2 days. (**c**) Location of the primers for confirming the genome structure of the *cna1* gene-deficient candidate by PCR using extracted genome DNA. (**d**) Confirmation of the genotypes of the *cna1* gene-deficient mutant (∆*cna1*) and its revertant (Rev.) by PCR using extracted genome DNA. (**e**–**h**) Generation of the *cna1* gene-deficient mutant and its revertant in *T. asahii*. (**e**) Strategy for generating the *cnb1* gene-deficient mutant (∆*cnb1*) and its revertant (Rev.). Predicted genomes of the *cnb1* gene-deficient mutant and its revertant are shown. (**f**) The parent strain (Parent), *cnb1* gene-deficient mutant (∆*cnb1*), and its revertant (Rev.) were spread on SDA with nourseothricin (Nou) (100 µg/ml) or hygromycin B (Hyg) (100 µg/ml) and incubated at 27 °C for 2 days. (**g**) Location of the primers for confirming the genome structure of the *cnb1* gene-deficient candidate by PCR using extracted genome DNA. (**h**) Confirmation of the genotypes of the *cnb1* gene-deficient mutant (∆*cnb1*) and its revertant (Rev.) by PCR using extracted genome DNA. Cropped blots were used. Full-length blots are presented in Supplementary Fig. S3.

The *ku70* gene-deficient strain of the *T. asahii* MPU129 strain has high homologous recombination efficiency, making it useful as a parent strain^28^. Using this strain, we generated the *cna1* gene-deficient mutant. The targeting DNA fragment used to generate the *cna1* gene-deficient mutant contains the *NAT1* gene, which leads to nourseothricin resistance (Fig. 1a). Introducing this DNA fragment into the *T. asahii* genome confers resistance to nourseothricin (Fig. 1a). Nourseothricin-resistant strains were obtained by introducing the DNA fragment through electroporation (Fig. 1b). The genomes were extracted from the transformants and the deficiency of the *cna1* gene was checked by polymerase chain reaction (PCR) (Fig. 1c,d). Secondary genetic mutations such as point mutations may occur during the generation of gene-deficient mutants. Therefore, it is necessary to establish a revertant strain in which the gene is reintroduced into the gene-deficient mutant to confirm that the phenotype of the gene-deficient strain is due to a deficiency of the targeted gene. Next, we generated a revertant strain of the *cna1* gene-deficient mutant. The targeting DNA fragment used to generate the revertant strain of the *cna1* gene-deficient mutant contains the *hgh* gene, which leads to hygromycin B resistance (Fig. 1a). Hygromycin B-resistant strains were obtained by introducing the DNA fragment through electroporation (Fig. 1b). Reintroduction of the *cna1* gene was checked by PCR using the genomes extracted from the transformants (Fig. 1c,d). The results confirmed the generation of the *cna1* gene-deficient *T. asahii* mutant and the *cna1* gene-reintroduced revertant. Similarly, a *cnb1* gene-deficient mutant and its revertant were also generated (Fig. 1e–h).

### Function of calcineurin in stress resistance of *T. asahii*

**Figure 2:**
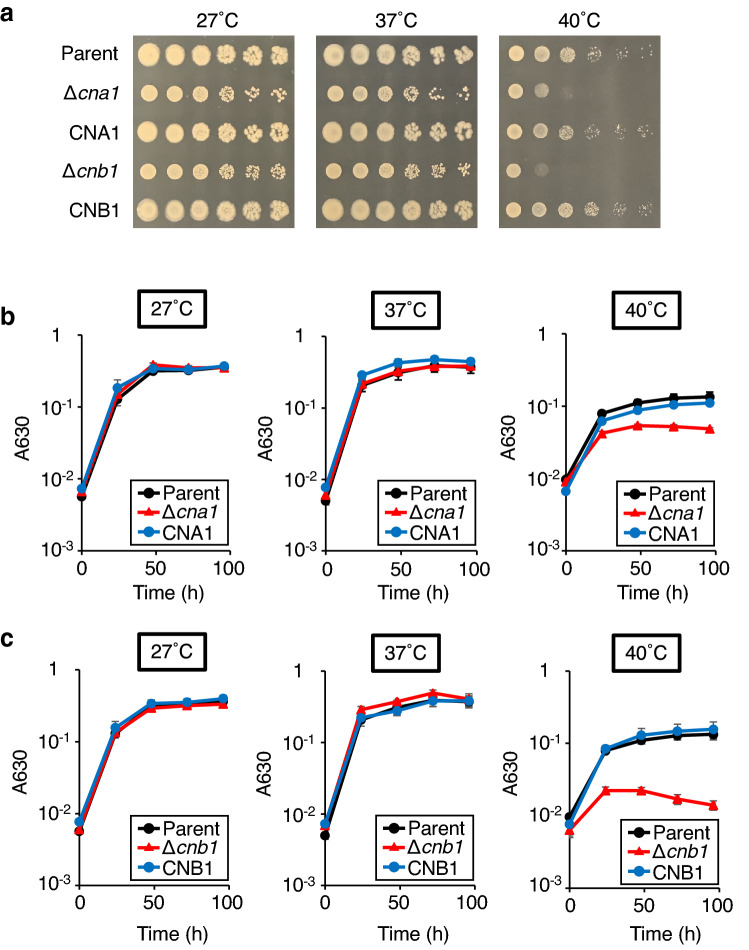
Temperature sensitivity of *cna1* or *cnb1* gene-deficiency in *T. asahii*. (**a**) The *T. asahii* parent strain (Parent), the *cna1* gene-deficient mutant (∆*cna1*), the revertant of ∆*cna1* (CNA1), the *cnb1* gene-deficient mutant (∆*cnb1*), and the revertant of ∆*cnb1* (CNB1) were grown on SDA and incubated at 27 °C for 2 days. *T. asahii* cells was suspended in physiologic saline solution and filtered through a 40-μm cell strainer. Series of tenfold dilution of the fungal suspension were prepared using saline. Five microliters of each cell suspension was spotted on the SDA. Agar plates were incubated at 27 °C, 37 °C, or 40 °C for 24 h. (**b**, **c**) The *T. asahii* parent strain (Parent), the *cna1* gene-deficient mutant (∆*cna1*), the revertant of ∆*cna1* (CNA1), the *cnb1* gene-deficient mutant (∆*cnb1*), and the revertant of ∆*cnb1* (CNB1) were inoculated on Sabouraud medium and incubated at 27 °C, 37 °C, or 40 °C. Absorbance of the culture at 630 nm was monitored. Data are shown as means ± standard error of the mean (SEM).

**Figure 3:**
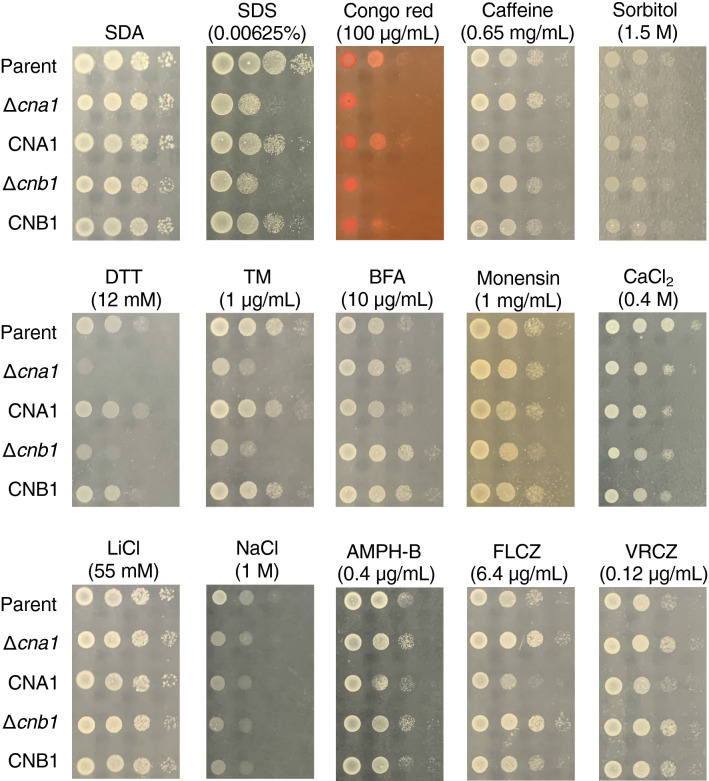
Sensitivity of the *cna1* and *cnb1* gene-deficient mutants against stress inducers. The *T. asahii* parent strain (Parent), the *cna1* gene-deficient mutant (∆*cna1*), the revertant of ∆*cna1* (CNA1), the *cnb1* gene-deficient mutant (∆*cnb1*), and the revertant of ∆*cnb1* (CNB1) were grown on SDA and incubated at 27 °C for 2 days. *T. asahii* cells was suspended in physiologic saline solution and filtered through a 40-μm cell strainer. Series of tenfold dilution of the fungal suspension were prepared using saline. Five microliters of each cell suspension was spotted on the SDA containing SDS (0.00625%), Congo red (100 µg/ml), caffeine (0.65 mg/ml), sorbitol (1.5 M), DTT (12 mM), TM (1 µg/ml), BFA (10 µg/ml), monensin (1 mg/ml), CaCl_2_ (0.4 M), LiCl (55 mM), NaCl (1 M), amphotericin-B (0.4 µg/ml), fluconazole (6.4 µg/ml), or voriconazole (0.12 µg/ml). Each agar plate was incubated at 37 °C for 24 h.

In *C. neoformans*, *cna1* and *cnb1* gene-deficient mutants show growth inhibition at 37 °C^29,30^. Growth of the *cna1* and *cnb1* gene-deficient *T. asahii* mutants was delayed at 40 °C (Fig. 2). The high-temperature sensitive phenotype of these gene-deficient mutants was suppressed in their revertants (Fig. 2). In *Cryptococcus gattii* and *C. neoformans*, *cna1* gene-deficient mutants are sensitive to sodium dodecyl sulfate (SDS), which damages the cell membrane, and Congo red, which damages the cell wall^21^. The *cna1* gene-deficient mutant of *C. neoformans* is also sensitive to caffeine, which inhibits the cell wall integrity (CWI) signaling pathway, which promotes cell wall integrity; and sorbitol, which is an osmotic stressor^22^. Growth was delayed in *cna1* and *cnb1* gene-deficient *T. asahii* mutants treated with SDS and Congo red, but not affected by treatment with caffeine or sorbitol (Fig. 3). In *C. neoformans*, the *cna1* gene-deficient mutant is sensitive to dithiothreitol (DTT) and tunicamycin (TM), which stress the ER; brefeldin A (BFA), which inhibits intracellular vesicle formation and protein trafficking between the ER and the Golgi apparatus; and monensin, which is involved in intracellular transport^22^. Growth was delayed in *cna1* and *cnb1* gene-deficient *T. asahii* mutants treated with DTT and TM, but not in those treated with BFA and monensin (Fig. 3). The presence of Ca^2+^ or Li^+^ inhibits the growth of the *cna1* gene-deficient *Cryptococcus gattii* mutant, but not that of the *cna1* gene-deficient *C. neoformans* mutant^21^. The growth of *cna1* and *cnb1* gene-deficient *T. asahii* mutants was not altered by treatment with CaCl_2_, LiCl, or NaCl (Fig. 3). Moreover, the presence of antifungal drugs such as amphotericin B, fluconazole, and voriconazole did not cause growth inhibition in the *cna1* and *cnb1* gene-deficient *T. asahii* mutants (Fig. 3). The stress-sensitive phenotypes of these gene-deficient mutants were suppressed in the revertants (Fig. 3). These findings suggest that the *cna1* and *cnb1* genes are involved in resistance to cell membrane and cell wall damage, and ER stress in *T. asahii*.

In *C. neoformans*, DTT induces the unfolded protein response (UPR) signaling^31^. The *HXL1* mRNA splicing by Ire1 protein, a key factor of UPR signaling, was increased by DTT in *C. neoformans*^31^. Moreover, the *HXL1* gene in *T. asahii* includes the putative unconventional splicing site that is spliced by Ire1 protein^32^. We tested the effect of DTT on the UPR signaling in *T. asahii*. The amounts of spliced *HXL1* mRNA in *T. asahii* were increased by treating DTT (Supplementary Fig. S1). Under DTT treatment, the amounts of spliced *HXL1* mRNA in the *cna1* and *cnb1* gene-deficient mutants were not altered compared to the parent strain (Supplementary Fig. S1). These results suggest that DTT induces the *HXL1* mRNA splicing independent to calcineurin signaling in *T. asahii*.

### Role of calcineurin in hyphal formation by *T. asahii*

**Figure 4:**
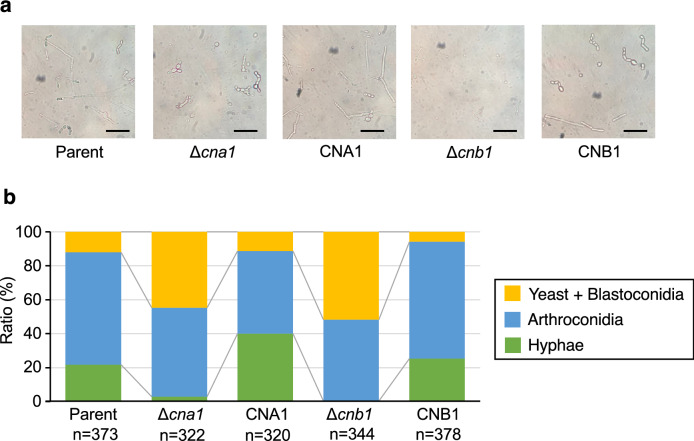
Effect of *cna1* or the *cnb1* gene-deficiency on *T. asahii* morphology in Sabouraud dextrose medium. The *T. asahii* parent strain (Parent), the *cna1* gene-deficient mutant (∆*cna1*), the revertant of ∆*cna1* (CNA1), the *cnb1* gene-deficient mutant (∆*cnb1*), and the revertant of ∆*cnb1* (CNB1) were grown on SDA and incubated at 27 °C for 2 days. *T. asahii* cells was suspended in physiologic saline solution and filtered through a 40-μm cell strainer. Absorbance at 630 nm of the *T. asahii* cell suspension was adjusted to 1–1.5. The cell suspension (10 µl) was added to Sabouraud dextrose medium (100 µl). The solutions were incubated at 37 °C for 48 h. The incubated solution was placed on glass slides and covered by a glass coverslip. (**a**) Samples were examined with bright light under a microscope. The scale bar indicated 50 µm. (**b**) The pictures were randomly obtained. The numbers of three cell types: yeast + blastoconidia, arthroconidia, and hyphae, were counted. The representative cells to determine the cell types are shown in Supplementary Fig. S2.

*Trichosporon asahii* has several morphologic forms: yeast, hyphae (filament form), and arthroconidia (chains of cells and asexual spores)^4^. In *Candida tropicalis*, calcineurin is a key factor that regulates to form hyphae^33^. We examined whether *cna1* and *cnb1* gene deficiencies affect hyphal formation in *T. asahii*. In Sabouraud dextrose medium, the hyphae ratio was lower in the *cna1* and *cnb1* gene-deficient mutants than in the parental strain (Fig. 4). The phenotype of lower hyphal forming activities in these gene-deficient mutants was suppressed in their revertants (Fig. 4). These observations suggest that the *cna1* and *cnb1* genes contribute to hyphal formation by *T. asahii*.

### Calcineurin-dependency of *T. asahii* virulence against silkworms

**Figure 5:**
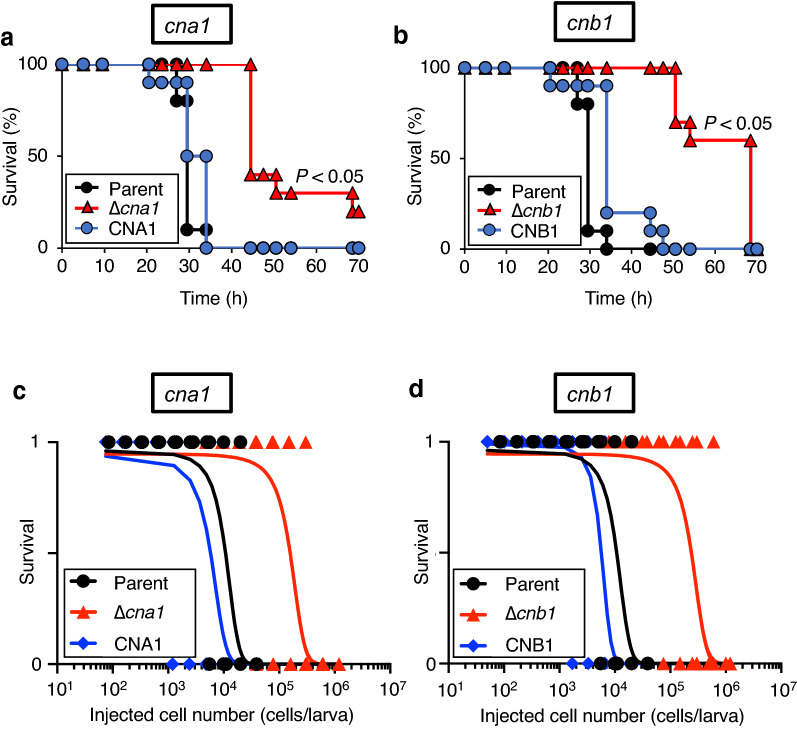
Attenuated pathogenicity in the *cna1* or the *cnb1* gene-deficient *T. asahii* mutants against silkworms. (**a**,**b**) The *T. asahii* parent strain (Parent; 2.9 × 10^5^ cells/larva), the *cna1* gene-deficient mutant (∆*cna1*; 7.4 × 10^5^ cells/larva), the revertant from ∆*cna1* (CNA1; 4.2 × 10^5^ cells/larva), the *cnb1* gene-deficient mutant (∆*cnb1*; 6.1 × 10^5^ cells/larva), or the revertant from ∆*cnb1* (CNB1; 7.1 × 10^5^ cells/larva) were injected into the silkworm hemolymph and the silkworms were incubated at 37 °C. Silkworm survival was monitored for 72 h. The significance of differences between the parent strain group and the *cnb1* gene-deficient mutant groups was calculated by the log-rank test based on the curves by the Kaplan–Meier method. *P* < 0.05 was considered significant. n = 10/group. (**c**,**d**) Number of surviving silkworms at conditions under 37 °C was determined at 48 h after administration of the fungal cells (50 to 1.2 × 10^6^ cells/larva) into the silkworm hemolymph. Surviving and dead silkworms are indicated as 1 and 0, respectively. n = 4/group. Curves were drawn from combined data of 2–3 independent experiments by a simple logistic regression model.

In *C. neoformans*, the *cna1* gene is required for virulence against silkworms^34^. We examined whether deficiencies of the *cna1* and *cnb1* genes reduced the virulence of *T. asahii* against silkworms. Survival times of silkworms injected with the *cna1* and *cnb1* gene-deficient mutants were longer than those of the parental strain (Fig. 5a,b). The half-maximal lethal dose (LD_50_) values of the *cna1* and *cnb1* gene-deficient mutants was more than tenfold higher that of the parent strain (Fig. 5c,d, and Table 1). These phenotypes were suppressed in their revertants (Fig. 5 and Table 1). These findings suggest that the *cna1* and *cnb1* genes are involved in the virulence of *T. asahii* against silkworms.

**Table 1:** The LD_50_ values of *T. asahii* strains.

*T. asahii* strains	LD_50_ (× 10^3^ cells/larva)
Parent strain	11
∆*cna1*	160
CNA1 (Revertant)	6
∆*cnb1*	240
CNB1 (Revertant)	6

### Calcineurin-dependency of *T. asahii* morphological change in silkworm hemolymph and human serum

**Figure 6:**
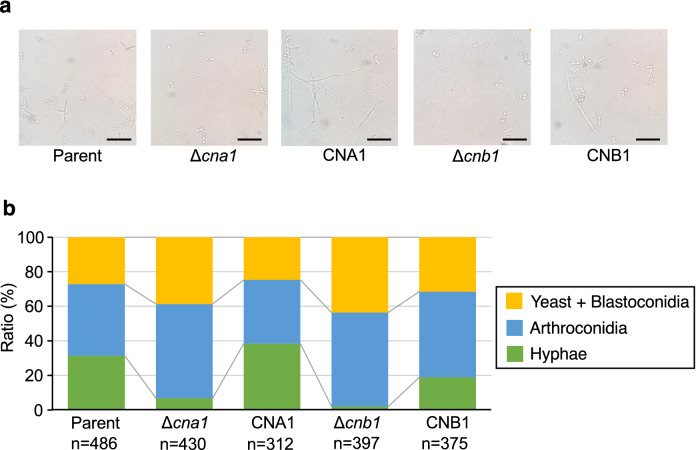
Low hyphal growth of *cna1* or the *cnb1* gene-deficient *T. asahii* mutants in harvested silkworm hemolymph. The *T. asahii* parent strain (Parent), the *cna1* gene-deficient mutant (∆*cna1*), the revertant of ∆*cna1* (CNA1), the *cnb1* gene-deficient mutant (∆*cnb1*), and the revertant of ∆*cnb1* (CNB1) were grown on SDA and incubated at 27 °C for 2 days. *T. asahii* cells was suspended in physiologic saline solution and filtered through a 40-μm cell strainer. Absorbance at 630 nm of the *T. asahii* cell suspension was adjusted to 1–1.5. The cell suspension (10 µl) was added to harvested silkworm hemolymph (100 µl) and the solutions were incubated at 37 °C for 48 h. The incubated solution was placed on glass slides and covered by a glass coverslip. (**a**) Samples were examined with bright light under a microscope. The scale bar indicated 50 µm. (**b**) The pictures were randomly obtained. The numbers of three cell types: yeast + blastoconidia, arthroconidia, and hyphae, were counted.

**Figure 7:**
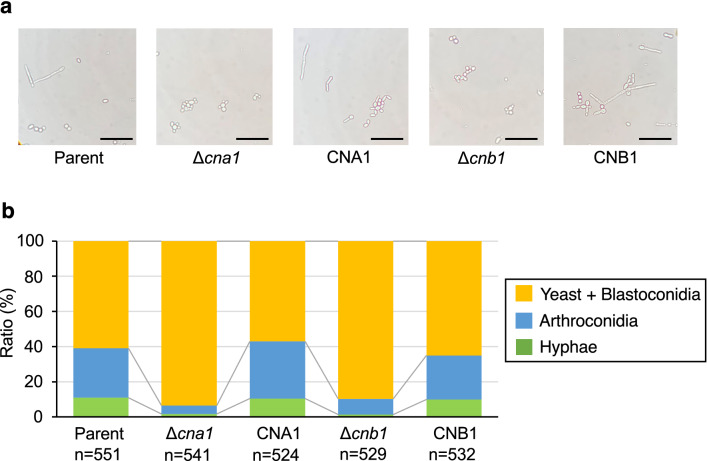
Effect of *cna1* or the *cnb1* gene-deficiency on *T. asahii* morphology in human serum. The *T. asahii* parent strain (Parent), the *cna1* gene-deficient mutant (∆*cna1*), the revertant of ∆*cna1* (CNA1), the *cnb1* gene-deficient mutant (∆*cnb1*), and the revertant of ∆*cnb1* (CNB1) were grown on SDA and incubated at 27 °C for 2 days. *T. asahii* cells was suspended in physiologic saline solution and filtered through a 40-μm cell strainer. Absorbance at 630 nm of the *T. asahii* cell suspension was adjusted to 1–1.5. The cell suspension (10 µl) was added to human serum (100 µl) and the solutions were incubated at 37 °C for 48 h. The incubated solution was placed on glass slides and covered by a glass coverslip. (**a**) Samples were examined with bright light under a microscope. The scale bar indicated 50 µm. (**b**) The pictures were randomly obtained. The numbers of three cell types: yeast + blastoconidia, arthroconidia, and hyphae, were counted.

We examined the effects of deficiencies of the *cna1* and *cnb1* genes on *T. asahii* morphology in the host environments. In the silkworm hemolymph, the hyphal ratio in the *cna1* and *cnb1* gene-deficient mutants was lower than that of the parent strain (Fig. 6). In human serum, the hyphae and arthroconidia ratio was also lower in the *cna1* and *cnb1* gene-deficient mutants than in the parental strain (Fig. 7). These phenotypes were suppressed in their revertants (Figs. 6 and 7). The results suggest that the *cna1* and *cnb1* genes are involved in the *T. asahii* morphological change in the host environments.

## Discussion

The present study using *cna1* and *cnb1* gene-deficient mutants demonstrated that calcineurin in *T. asahii* plays a vital role in stress resistance and virulence against silkworms. Our findings suggest that the pathogenicity of *T. asahii* is regulated via the calcineurin-signaling pathway.

The calcineurin-signaling pathway is required for normal growth of *T. asahii* under a high-temperature condition (40 °C). Severe growth inhibition of the *cna1* and *cnb1* gene-deficient *T. asahii* mutants were observed at 40 °C compared with those at 27 °C and 37 °C. In *C. neoformans*, calcineurin is required for the growth at 37 °C^29,30^. Therefore, the high-temperature stress response is partially regulated by calcineurin in *T. asahii* compared with *C. neoformans*.

In *Cryptococcus gattii* and *C. neoformans*, calcineurin plays an important role in cell membrane homeostasis, cell wall integrity, the ER stress response, cation homeostasis, and fluconazole tolerance^21,22^. Calcineurin in *T. asahii* is related to resistance against cell membrane and cell wall damage, and ER stress, but not cell wall integrity dependent on the CWI pathway, osmotic stress resistance, cation homeostasis, or antifungal drug resistance. The *cna1* gene-deficient mutant of *C. neoformans* exhibits sensitivities to SDS, Congo red, caffeine, sorbitol, BFA, monensin, DTT, TM, CaCl_2_, LiCl, NaCl, and fluconazole^21,22,35,36^. Therefore, the roles of calcineurin in cell wall integrity dependent on the CWI pathway, osmotic stress resistance, cation homeostasis, and antifungal drug resistance differ between *T. asahii* and *C. neoformans*. Cheon and colleagues reported that DTT induces the Ire1-mediated UPR signaling in *C. neoformans*^31^. In *T. asahii*, DTT also induced the *HXL1* mRNA splicing by Ire1 protein. The *HXL1* mRNA splicing induced by DTT occurred in the *cna1* and *cnb1* gene-deficient *T. asahii* mutants. Therefore, calcineurin does not regulate Ire1-mediated UPR signaling caused by DTT in *T. asahii*, at least in the experimental condition. Revealing the molecular mechanisms of calcineurin on the stress response in *T. asahii* is an important future subject.

*Trichosporon asahii* forms hyphae in Sabouraud dextrose medium and in silkworm hemolymph via calcineurin. Moreover, calcineurin is required for *T. asahii* virulence in a silkworm infection model. In *Candida dubliniensis*, *Candida tropicalis*, and *Aspergillus fumigatus*, the calcineurin signaling pathway regulates hyphal growth and virulence^37,38^. *T. asahii* forms hyphae in the hemolymph of silkworms infected with *T. asahii*^23^. Hyphal growth of *T. asahii* in blood vessels causes necrotic thrombi and may contribute to infection^39^. Moreover, we demonstrated that the calcineurin contributes to arthroconidia and hyphal formation of *T. asahii* in human serum. Therefore, morphological change of *T. asahii* regulated by calcineurin might contribute to its virulence against silkworms and humans. Further studies are needed to reveal the role of the calcineurin in *T. asahii* virulence using a mouse infection model. The calcineurin in *C. neoformans* regulates gene expression via dephosphorylation of the transcription factors^40^. We speculate that the calcineurin in *T. asahii* regulates hyphal formation-related gene expression. Identifying the crucial genes involved in hyphal formation regulated by calcineurin in *T. asahii* will be an important next step.

Tacrolimus (FK506), a calcineurin inhibitor, affects the physiologies of several pathogenic fungi^41,42^. On the other hand, FK506 suppresses the immune system by blocking T-cell activation in human^43^. Therefore, the immunosuppressive effect should be eliminated when used as a treatment for fungal infections. Juvvadi and colleagues succeeded to develop the low immunosuppressive FK506 analog APX879 that inhibits the *Aspergillus fumigatus* calcineurin^44^. We assumed that the fungal calcineurin specific inhibitors might be effective against *T. asahii* infection.

In conclusion, calcineurin in *T. asahii* has roles in the stress response, hyphal formation, and virulence. We assumed that calcineurin is a potent target for anti-infectious agents against *T. asahii* infection.

## Methods

### Reagents

SDS, sorbitol, DTT, NaCl, and LiCl were purchased from Wako Pure Chemical Industries (Osaka, Japan). Fluconazole, voriconazole, hygromycin B, caffeine, and monensin sodium salt were purchased from Tokyo Chemical Industry Co., Ltd. (Tokyo, Japan). Nourseothricin was purchased from Jena Bioscience (Dortmund, Germany). Congo red was purchased from Sigma-Aldrich (St. Louis, MO, USA). G418 was purchased from Enzo Life Science, Inc. (Farmingdale, NY, USA). CaCl_2_ was purchased from Kanto Chemical Co., Inc. (Tokyo, Japan). Tunicamycin was purchased from Cayman Chemical Company (Ann Arbor, MI, USA). Brefeldin A (BFA) was purchased from Funakoshi Co., Ltd. (Tokyo, Japan). Human serum [(from male AB clotted whole blood), USA origin, sterile-filtered] (Product ID: H6914) was purchased from Sigma-Aldrich (St. Louis, MO, USA).

### Culture of *T. asahii*

The *T. asahii* strain (MPU129 *ku70* gene-deficient mutant) used in this study was generated as previously reported^28^. The *T. asahii* MPU129 *ku70* gene-deficient mutant was grown on Sabouraud dextrose agar (SDA) (1% hipolypepton [Nihon Pharmaceutical Co., Ltd., Tokyo, Japan], 4% dextrose, and 1.5% agar [both from FUJIFILM Wako Pure Chemical Industries, Osaka, Japan]) containing G418 (50 μg/ml) and incubated at 27 °C for 2 days.

### Construction of gene-deficient *T. asahii* mutants and their revertants

The plasmid for gene-deficient *T. asahii* strains was constructed according to a previous report^45^. To generate the *cna1* or *cnb1* gene-deficient strains, the 5’-UTR and 3’-UTR of the *cna1* or *cnb1* gene were introduced into a pAg1-NAT1 vector^46^. To generate their revertants, the hygromycin phosphotransferase gene (*hph*) cassette and the *cna1* or *cnb1* gene were introduced into pAg1-*cna1*(5’UTR)-*NAT1*-*cna1*(3’UTR) or pAg1-*cnb1*(5’UTR)-*NAT1*-*cnb1*(3’UTR). Cloning was performed by the infusion method (In-Fusion HD Cloning Kit, Takara, Shiga, Japan) or ligation method (DNA Ligation Kit Ver.2.1, Takara, Shiga, Japan). The primers used for PCR amplification of each DNA region are shown in Supplementary Table S1.

Competent cells for electroporation were prepared according to a previous report^28^. *T. asahii* was spread on an SDA plate and cultured at 27 °C for 1 day. *T. asahii* cells on agar were suspended by physiologic saline solution (2 ml), and the suspension was transferred to a 1.5-ml tube. The fungal cells were collected by centrifugation at 8000 rpm for 3 min (TOMY-MX100, TOMY Digital Biology Co. Ltd, Tokyo, Japan) and suspended by adding 1 ml of ice-cold water and centrifuged at 8000 rpm for 3 min. This washing process was repeated 4 times. The washed cells were suspended by adding 1 ml of 1.2 M sorbitol solution and centrifuged at 8000 rpm for 3 min. The obtained fungal cells were suspended with 0.2 ml of 1.2 M sorbitol solution as competent cells. The PCR-amplified 5'-UTR (*cna1*) -*NAT1*-3'-UTR (*cna1*), 5'-UTR (*cna1*) -*cna1*-*hph*-3'-UTR (*cna1*), 5'-UTR (*cnb1*) -*NAT1*-3'-UTR (*cnb1*), or 5'-UTR (*cnb1*) -*cnb1*-*hph*-3'-UTR (*cnb1*) fragments (180 ng/2 µl) were added to the *T. asahii* competent cells (40 µl) and placed on ice for 15 min. The suspension was added to a 0.2-cm gap cuvette (Bio-Rad Laboratories, Inc.) and electroporated (Time constant protocol: 1800 V, 5 ms) using a Gene Pulser Xcell (Bio-Rad Laboratories, Inc.). The cells were suspended by adding 500 µl YPD containing 0.6 M sorbitol and incubated at 27 °C for 3 h. After incubation, the cells were collected by centrifugation at 10,000 rpm for 5 min and suspended in 100 µl of physiologic saline solution and applied to SDA containing nourseothricin (300 µg/ml) or hygromycin B (300 µg/ml). The cells were incubated at 27 °C for 3 days and the growing colonies were isolated as gene-deficient strain or revertant candidates. Introduction of the mutation into the genome of the candidate strains was confirmed using the extracted genome as a template DNA by PCR with the primers shown in Supplementary Table S1. The information of strains were shown in Table 2.

**Table 2:** *T. asahii* strains used in this study.

*T. asahii* strains	Relevant genotype	Background	Reference
MPU129 ∆*ku70* (Parent strain)	*ku70*::*nptII*	MPU129	Matsumoto et al.^28^
∆*cna1*	*ku70*::*nptII*, *cna1*::*NAT1*	MPU129 ∆*ku70*	This study
CNA1 (Revertant)	*ku70*::*nptII*, *NAT1*::*cna1*, *hgh*	MPU129 ∆*ku70* ∆*cna1*	This study
∆*cnb1*	*ku70*::*nptII*, *cnb1*::*NAT1*	MPU129 ∆*ku70*	This study
CNB1 (Revertant)	*ku70*::*nptII*, *NAT1*::*cnb1*, *hgh*	MPU129 ∆*ku70* ∆*cnb1*	This study

### Temperature sensitivity test

The *T. asahii* strains were grown on SDA containing G418 (50 μg/ml) and incubated at 27 °C for 2 days. *T. asahii* cells was suspended in physiologic saline solution (0.9% w/v NaCl) and filtered through a 40-μm cell strainer (Corning Inc., Corning, NY, USA). Absorbance of 630 nm of the *T. asahii* cell suspension was adjusted to 1. Series of tenfold dilution of the fungal suspension were prepared using saline. Five microliters of each cell suspension was spotted on the SDA. The agar plates were incubated at 27, 37, or 40 °C for 24 h, and photographs were obtained.

For growth on liquid medium, Sabouraud liquid medium (1% hipolypepton, 4% dextrose) was used in this study. Suspensions of the *T. asahii* parent strain (Parent) the *cna1* gene-deficient mutant (∆*cna1*), the revertant from ∆*cna1* (CNA1), the *cnb1* gene-deficient mutant (∆*cnb1*), and the revertant from ∆*cnb1* (CNB1) were prepared with Sabouraud medium and adjusted to 0.005 on absorbance at 630 nm. The *T. asahii* suspensions were incubated at 27 °C, 37 °C, or 40 °C for 4 days and absorbance at 630 nm was measured using a microplate reader (iMark™ microplate reader; Bio-Rad Laboratories Inc., Hercules, CA, USA).

### Drug sensitivity test

The *T. asahii* strains were grown on SDA containing G418 (50 μg/ml) and incubated at 27 °C for 2 days. *T. asahii* cells was suspended in physiologic saline solution (0.9% w/v NaCl) and filtered through a 40-μm cell strainer (Corning Inc.). Absorbance of 630 nm of the *T. asahii* cell suspension was adjusted to 1. Series of tenfold dilution of the fungal suspension were prepared using saline. Five microliters of each cell suspension was spotted on the SDA containing SDS (0.00625%), Congo red (100 µg/ml), caffeine (0.65 mg/ml), sorbitol (1.5 M), DTT (12 mM), TM (1 µg/ml), BFA (10 µg/ml), Monensin (1 mg/ml), CaCl_2_ (0.4 M), LiCl (55 mM), NaCl (1 M), amphotericin B (0.4 µg/ml), fluconazole (6.4 µg/ml), or voriconazole (0.12 µg/ml). Each agar plate was incubated at 37 °C for 24 h, and photographs were obtained.

### Observation of *T. asahii* morphology

The *T. asahii* strains were grown on SDA containing G418 (50 μg/ml) and incubated at 27 °C for 2 days. *T. asahii* cells was suspended in physiologic saline solution and filtered through a 40-μm cell strainer (Corning Inc.). Absorbance of 630 nm of the *T. asahii* cell suspension was adjusted to 1–1.5. The cell suspension (10 µl) was added to Sabouraud dextrose medium, harvested silkworm hemolymph, or human serum (100 µl). The solutions were incubated at 37 °C for 48 h. The incubated solution was placed on glass slides and covered by a glass coverslip. Samples were examined with bright light under a microscope (CH-30; Olympus, Tokyo, Japan). The pictures were randomly obtained. The morphology of *T. asahii* was determined according to a previous report^15^. The numbers of the 3 types of cells, yeast, arthroconidia, and hyphae, were counted.

### Silkworm infection experiments

Silkworm infection experiments were performed according to a previous report^23^. Eggs of silkworms (Hu Yo × Tukuba Ne) were purchased from Ehime-Sanshu Co, Ltd. (Ehime, Japan), disinfected, and hatched at 25–27 °C. The silkworms were fed an artificial diet, Silkmate 2S, containing antibiotics purchased from Ehime-Sanshu Co., Ltd. Fifth instar larvae were used in the infection experiments. Silkworm fifth instar larvae were fed the artificial diet (Silkmate 2S; Ehime-Sanshu Co., Ltd.) overnight. *T. asahii* grown on SDA plates was suspended in physiologic saline solution (0.9% w/v NaCl) and filtered through a 40-μm cell strainer (Corning Inc.). A 50-µl suspension of *T. asahii* cells was administered to the silkworm hemolymph by injecting the silkworm dorsally using a 1-ml tuberculin syringe (Terumo Medical Corporation, Tokyo, Japan). Silkworms injected with *T. asahii* cells were placed in an incubator and their survival was monitored.

### LD_50_ measurement

The dose of *T. asahii* required to kill half of the silkworms (LD_50_) was determined according to the previous report^23^. *T. asahii* strains (1 × 10^2^ to 2 × 10^7^ cells/50 µl) were injected into the silkworm hemolymph and the silkworms were incubated at 37 °C. Survival of the silkworms (n = 4/group) at 48 h was recorded. The LD_50_ was determined from the combined data of 2–3 independent experiments by a simple logistic regression model using Prism 9.1.2 (GraphPad Software, LLC, San Diego, CA, USA, https://www.graph pad.com/scientific -software/prism/).

### Statistical analysis

All experiments were performed at least twice and representative results are shown. The significance of differences between groups in the silkworm infection experiments was calculated by the log-rank test based on curves determined by the Kaplan–Meier method using Prism 9.1.2. *P* < 0.05 was considered significant.

## Supplementary Information

Supplementary Information.

## Data Availability

The datasets generated during the present study are available from the corresponding author on reasonable request.
