# Supplementary material for: A critical role of calcineurin in stress responses, hyphal formation, and virulence of the pathogenic fungus Trichosporon asahii
Source: Sci Rep. 2022 Sep 27;12:16126. doi: 10.1038/s41598-022-20507-x (PMC9515189; doi:10.1038/s41598-022-20507-x)
Supplement: Supplementary file 1 — Supplementary Information. [file 41598_2022_20507_MOESM1_ESM.pdf]

Supplementary information

A critical role of calcineurin in stress responses, hyphal formation, and virulence of the pathogenic fungus *Trichosporon asahii*

Yasuhiko Matsumoto[1*], Asami Yoshikawa[1], Tae Nagamachi[1], Yu Sugiyama[1], Tsuyoshi Yamada[2,3], and Takashi Sugita[1]

[1]Department of Microbiology, Meiji Pharmaceutical University, 2-522-1, Noshio, Kiyose, Tokyo 204-8588, Japan.

[2]Teikyo University Institute of Medical Mycology, 359 Otsuka, Hachioji, Tokyo 192-0395, Japan.

[3]Asia International Institute of Infectious Disease Control, Teikyo University, 2-11-1, Kaga, Itabashi-ku, Tokyo, 173-8605, Japan

*Address correspondence to: Dr. Yasuhiko Matsumoto, Department of Microbiology, Meiji Pharmaceutical University, 2-522-1, Noshio, Kiyose, Tokyo 204-8588, Japan, Tel: +81-42-495-8745, e-mail: ymatsumoto@my-pharm.ac.jp.

Supplementary table 1 Primers used in this study

Primers	Nucleic acid sequence
pAg1-*cna1*(5'UTR)-*NAT1*-*cna1*(3'UTR) for cloning	
**pAg1-*cna1* (5'UTR)-*NAT1* (1st cloning)**	
F pAg1 for *cna1*(5'UTR)	CTCCGCTCATGATCAGATTGTCGTTTCCCG
R *NAT1* for *cna1*(5'UTR)	GCTGCGAGGATGTGAGCTGGAGAGC
F *cna1*(5'UTR)	ATCTGATCATGAGCGGAGCGAGTTGCCAAACTG CTGGCGAAGCTTGG
R *cna1*(5'UTR)	AGCTCACATCCTCGCAGCCCTTGGTGCGCTGTCGGTCGATGTTCGC
**pAg1-*cna1*(5'UTR)-*NAT1*-*cna1*(3'UTR) (2nd cloning)**	
F pAg1 for *cna1*(3'UTR)	GAAAACCCTGGCGTTACCCAACTTAATCG
R *NAT1* for *cna1*(3'UTR)	GAAGAGATGTAGAAACTAGCTTCCTGGTTTCAGAG
F *cna1*(3'UTR)	TAGTTTCTACATCTCTTCCGGCTCGTCGGTGTTGGCAAAATTGTGGTCG
R *cna1*(3'UTR)	GGTAACGCCAGGGTTTTCGCCTTCTGGCTCGGATGCCTCTCCAAGACC
Amplification of *cna1* cassette for electroporation	
F *cna1*-cassette	ATCTGATCATGAGCGGAGCGAGTTGCCAAACTGCTGGCGAAGCTTGG
R *cna1*-cassette	GGTAACGCCAGGGTTTTCGCCTTCTGGCTCGGATGCCTCTCCAAGACC
pAg1-*cna1*(5'UTR)-*cna1*-*hph*-*cna1*(3'UTR) for cloning	
**pAg1-*cna1*(5'UTR)-*hph*-*cna1*(3'UTR) (1st cloning)**	
F 3'UTR-pAg1-5'UTR for *hph*	CGGCTCGTCGGTGTTGGCAAAATTGTGGTCG
R 3'UTR-pAg1-5'UTR for *hph*	CCTTGGTGCGCTGTCGGTCGATGTTCGC
F *hph*	ACCGACAGCGCACCAAGGGGGCCCCCTGCGAGGATG
R *hph*	GCCAACACCGACGAGCCGGGATCCGAAGAGATGTAGAAAC
**pAg1-*cna1*(5'UTR)-*cna1*-*hph*-*cna1*(3'UTR) (2nd cloning)**	
F *hph*-3'UTR-pAg1-5'UTR for *cna1*	GGGCCCCCTGCGAGGATG
R *hph*-3'UTR-pAg1-5'UTR for *cna1*	CCTTGGTGCGCTGTCGGTCGATGTTCGC
F *cna1*	ACCGACAGCGCACCAAGG ATGACCTCGCCTACCACCCAAACCGCC
R *cna1*	CATCCTCGCAGGGGGCCC CTTAGGCAATCGAGTTCTCCGGACGCG
Amplification of *cna1* revertant cassette for	

electroporation	
F *cna1* revertant-cassette	ATCTGATCATGAGCGGAGCGAGTTGCCAAACTGCTGGCGAAGCTTGG
R *cna1* revertant-cassette	GGTAACGCCAGGGTTTTCGCCTTCTGGCTCGGATGCCTCTCCAAGACC
**Primers-1 for *cna1* genotyping**	
F *cna1* gene locus	GCGTATGCACGCACGCACCTTCCTGGG
R *cna1* gene locus	AGTCTCCCCCTCCACCAACCATCCGACCAC
**Primers-2 for *cna1* genotyping**	
F *cna1* gene ORF	GCGAGGGACGATTGACGGAGGAGCAGGC
R *cna1* gene ORF	GACGACACTCGTGGTTGCCTCGAAGCAGG
**Primers-3 for *cna1* genotyping**	
F *cna1* gene outside 1	CACCGAGTGCCAGCTGCATGTTAGCTACCGC
R *cna1* gene outside 1	CTGGTGCGGTACCGGTAAGCCGTGTCGTCAAG
**Primers-4 for *cna1* genotyping**	
F *cna1* gene outside 2	GGACGGCGAGCAGGCGCTCTACATGAGC
R *cna1* gene outside 2	GGCGATTACGTACGGCGTCTTCGAGCGCG
**Primers-5 for *cna1* genotyping**	
F *cna1* gene outside 1	CACCGAGTGCCAGCTGCATGTTAGCTACCGC
R *cna1* gene outside 3	CCGAGAGCTGCATCAGGTCGGAGACGC
**Primers-6 for *cna1* genotyping**	
F *cna1* gene outside 3	CAGGGTCGATGCGACGCAATCGTCCGATCC
R *cna1* gene outside 2	GGCGATTACGTACGGCGTCTTCGAGCGCG
pAg1-*cnb1*(5'UTR)-*NAT1*-*cnb1*(3'UTR) for cloning	
F *cnb1* (5'UTR)	TGAACTAGTCCGTGATCTGCTGCACGTTCGGGTCC
R *cnb1* (5'UTR)	AAAGGGCCCAAGATCTAGTGATAGATGTGTGGAGA
F *cnb1* (3'UTR)	CTGGGATCCGCGCGCACACACGGATGTGAGCGTAA
R *cnb1* (3'UTR)	CGCGGTACCACTGTTCACCTCTGGCATTGTTACGA
Amplification of *cna1* cassette for electroporation	
F *cnb1*-cassette	CCGTGATCTGCTGCACGTTCGGGTCCG
R *cnb1*-cassette	CTGTTCACCTCTGGCTACGACCCCCTCCTC
pAg1-*cnb1*(5'UTR)-*cnb1*-*hph*-*cnb1*(3'UTR) for cloning	

***cnb1* 5'-UTR/ORF fragment**	
**1st PCR**	
TaCNA-F1/SpeI	TGAACTAGTCCGTGATCTGCTGCACGTTCGGGTCC
TaCNB-R2(-KpnI)	GTGCGTGCGCGGTACTCACGATCGGTGTAATAGC
TaCNB-F2(-KpnI)	GCTATTACACCGATCGTGAGTACCGCGCACGCAC
TaCNB-R6/ApaI	TACGCTCACATCCGTGTTTGGGCCCTTAGAACAGG
**2nd PCR (overlapping PCR)**	
TaCNA-F1/SpeI	TGAACTAGTCCGTGATCTGCTGCACGTTCGGGTCC
TaCNB-R6/ApaI	TACGCTCACATCCGTGTTTGGGCCCTTAGAACAGG
***hph* cassette**	
**1st PCR**	
CnPactin(F)ApaI	TTTGGGCCCCCTGCGAGGATGTGAGCTGGAGAGCG
CnPact-hph(R)	GAGTTCAGGCTTTTTCATACGCGTAGACATGTTGG
CnPact-hph(F)	CCAACATGTCTACGCGTATGAAAAAGCCTGAACTC
hph-Ttrp1(R)	ACCGCCTTCACGAATTCCTATTCCTTTGCCCTCGG
hph-Ttrp1(F)	CCGAGGGCAAAGGAATAGGAATTCGTGAAGGCGGT
Ttrp1(R)BamHI	AAAGGATCCGAAGAGATGTAGAAACTAGC
**2nd PCR (overlapping PCR)**	
CnPactin(F)ApaI	TTTGGGCCCCCTGCGAGGATGTGAGCTGGAGAGCG
Ttrp1(R)BamHI	AAAGGATCCGAAGAGATGTAGAAACTAGC
**Primers-1 for *cnb1* genotyping**	
F *cnb1* gene locus	GGAGTGAAGAAGGGCAGAGAGCAACAACAGCGGT
R *cnb1* gene locus	CCGTGATCGCATGGGGCGTGCACAAAGTG
**Primers-2 for *cnb1* genotyping**	
F *cnb1* gene ORF	CGGCTCGGGTACGGTAGACTTCCAGGAGTTTGTCG
R *cnb1* gene ORF	AACAGGTCCTCGAGCGTCATCTGCTTGACGATGT
**Primers-3 for *cnb1* genotyping**	
F *cnb1* gene outside 1	CATATCCCTCACGTTGCGGTCGAGCGCC
R *cnb1* gene outside 1	CTGGTGCGGTACCGGTAAGCCGTGTCGTCAAG

**Primers-4 for *cnb1* genotyping**	
F *cnb1* gene outside 2	GGACGGCGAGCAGGCGCTCTACATGAGC
R *cnb1* gene outside 2	CTGAGTCCCATCGGCCCTTGCCTTCAAGCTACC
**Primers-5 for *cnb1* genotyping**	
F *cnb1* gene outside 1	CATATCCCTCACGTTGCGGTCGAGCGCC
R *cnb1* gene outside 3	CCGAGAGCTGCATCAGGTCGGAGACGC
**Primers-6 for *cnb1* genotyping**	
F *cnb1* gene outside 3	CAGGGTCGATGCGACGCAATCGTCCGATCC
R *cnb1* gene outside 2	CTGAGTCCCATCGGCCCTTGCCTTCAAGCTACC
**RT-primers-1 for RT-PCR**	
F*HXL1*	AACGCTCACCTCCGCTGGCGCG
R*HXL1* (intact)	GGGGGCAGGCGACGAGATGGGAGTGC
**RT-primers-2 for RT-PCR**	
F*HXL1*	AACGCTCACCTCCGCTGGCGCG
R*HXL1* (Splicing)	GCTGGCAAGTCCCAGTTGATTCCTGTCAAAG

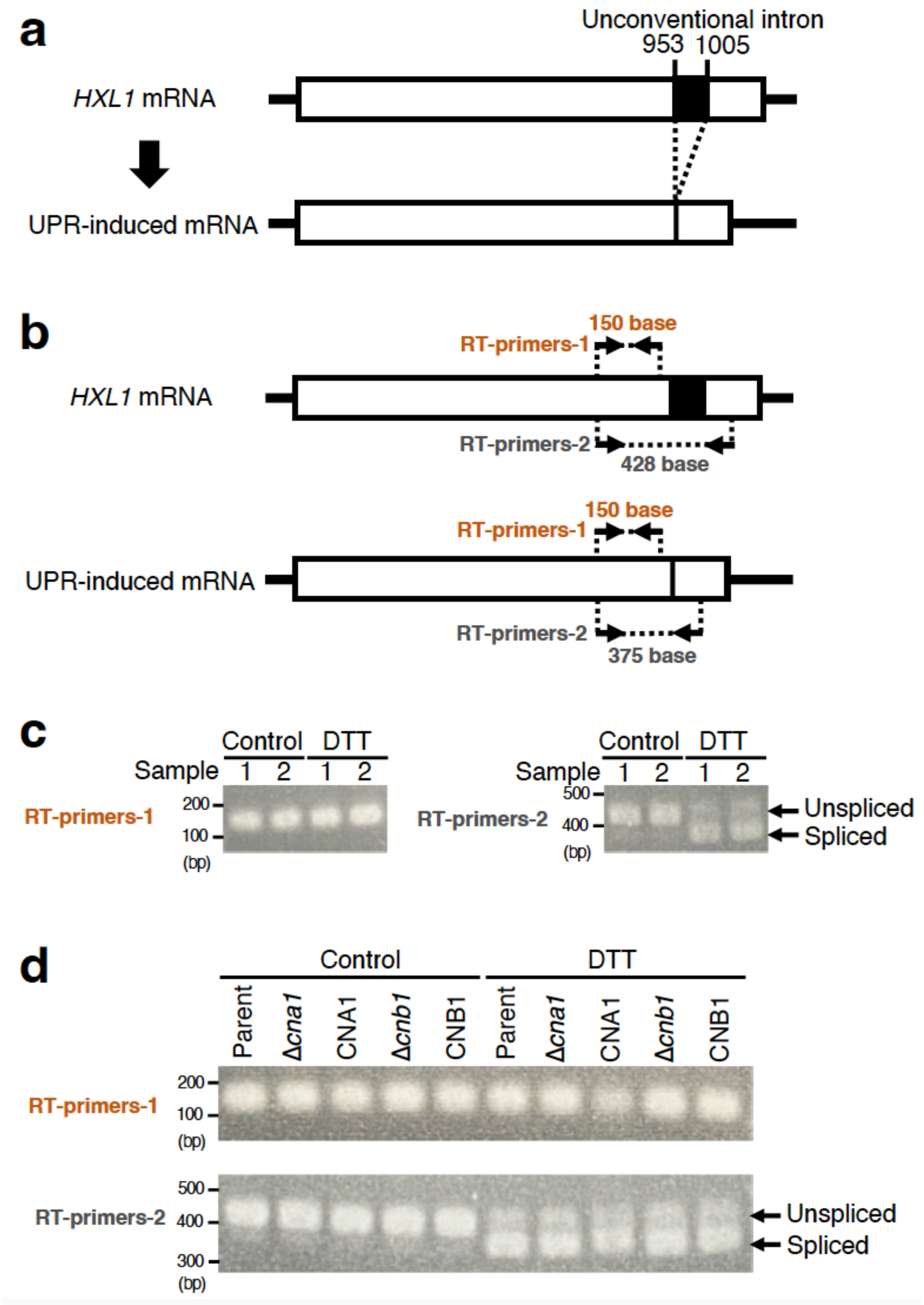
a
Unconventional intron
953 1005
HXL1 mRNA
UPR-induced mRNA
b
150 base
RT-primers-1
HXL1 mRNA
RT-primers-2
428 base
150 base
RT-primers-1
UPR-induced mRNA
RT-primers-2
375 base
c
Control DTT
Sample 1 2 1 2
RT-primers-1
200
100
(bp)
Control DTT
Sample 1 2 1 2
RT-primers-2
500
400
(bp)
Unspliced
Spliced
d
Control
DTT
Parent
Δcna1
CNA1
Δcnb1
CNB1
Parent
Δcna1
CNA1
Δcnb1
CNB1
RT-primers-1
200
100
(bp)
RT-primers-2
500
400
300
(bp)
Unspliced
Spliced

Supplementary Fig. S1 Effect of DTT on the *HXL1* mRNA splicing in *T. asahii.*
(**a**) The *HXL1* mRNA splicing by Ire1-mediated UPR signaling in *T. asahii*. (**b**) Location of the primers for RT-PCR. (**c**) The *T. asahii* parent strain was incubated on Sabouraud dextrose medium with or without DTT (12 mM) at 37°C for 1 h. Total RNA was extracted according to the general method (NucleoSpin RNA, Takara, Shiga, Japan). Reverse transcription was performed using a kit (ReverTra Ace qPCR RT Master Mix, TOYOBO Co., Ltd., Osaka, Japan). Primers used in the experiments were listed in Supplementary table 1. Control: without DTT. DTT: with DTT (12 mM). Two samples per group were shown. (**d**) The *T. asahii* parent strain (Parent), the *cna1* gene-deficient mutant (Δ*cna1*), the revertant of Δ*cna1* (CNA1), the *cnb1* gene-deficient mutant (Δ*cnb1*), and the revertant of Δ*cnb1* (CNB1) was incubated on Sabouraud dextrose medium with or without DTT (12 mM) at 37°C for 1 h. Control: without DTT. DTT: with DTT (12 mM).

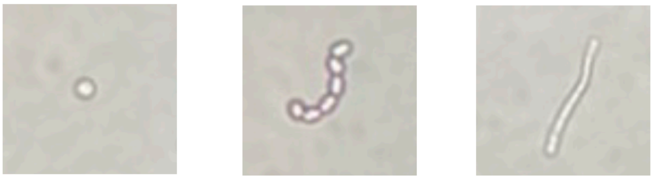

Supplementary Fig. S2 The representative cells to determine the cell types.

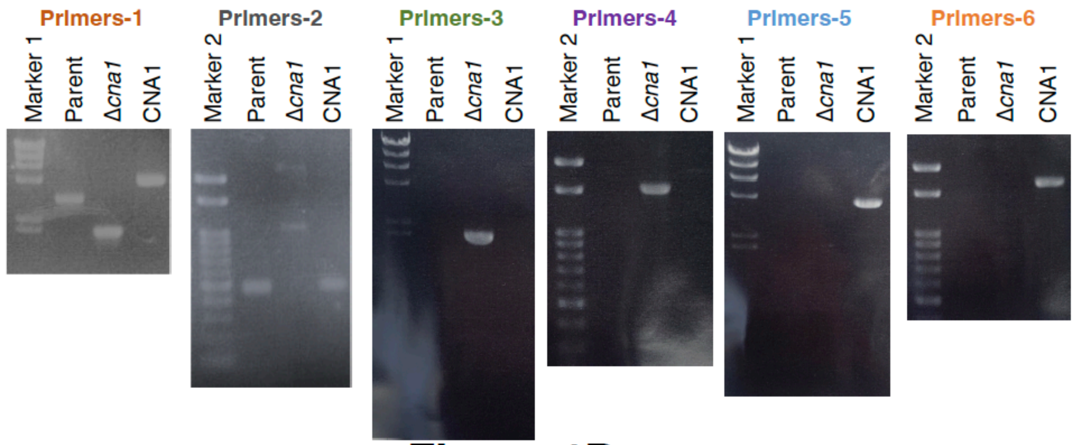

Figure 1D

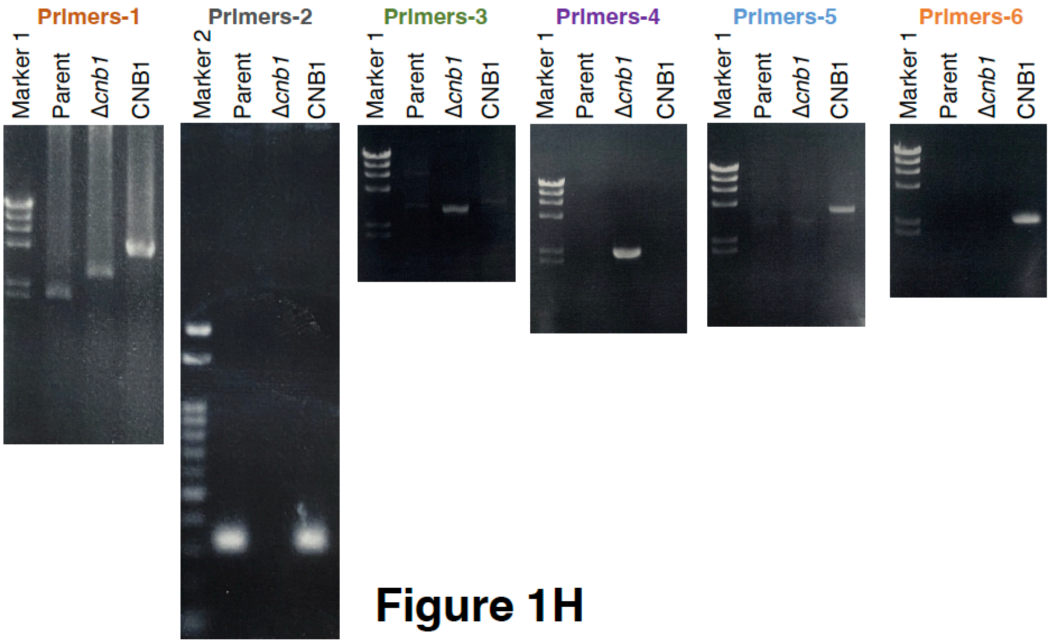

Figure 1H

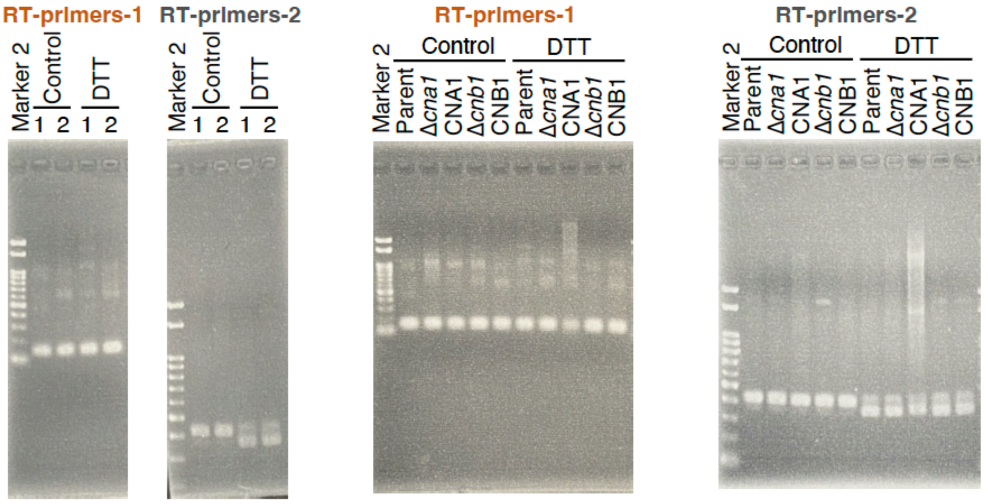

Supplementary Fig. S1

Supplementary Fig. S3 Full-length blots of Figure 1d and 1h. Marker 1: OneSTEP Marker 1(λ /HindIII digest) (Nippon Gene Co., Ltd., Tokyo, Japan), Maker 2: Gene Ladder 100 (Nippon Gene Co., Ltd., Tokyo, Japan).
